# Accelerated Biodegradation of the Agrochemical Ametoctradin by Soil-Derived Microbial Consortia

**DOI:** 10.3389/fmicb.2020.01898

**Published:** 2020-08-25

**Authors:** Hunter D. Whittington, Mahatam Singh, Chanh Ta, M. Andrea Azcárate-Peril, José M. Bruno-Bárcena

**Affiliations:** ^1^Department of Plant and Microbial Biology, North Carolina State University, Raleigh, NC, United States; ^2^BASF Corporation, Research Triangle Park, NC, United States; ^3^Department of Medicine, Division of Gastroenterology and Hepatology, and UNC Microbiome Core, Center for Gastrointestinal Biology and Disease, School of Medicine, University of North Carolina at Chapel Hill, Chapel Hill, NC, United States

**Keywords:** biodegradation, fungicide, ametoctradin, microbial consortium, packed-bed reactor

## Abstract

Pesticide-resistant plant pathogens are an increasing threat to the global food supply and have generated a need for novel, efficacious agrochemicals. The current regulatory process for approving new agrochemicals is a tedious but necessary process. One way to accelerate the safety evaluation process is to utilize *in vitro* systems to demonstrate pesticide degradation by soil microbes prior to *ex vivo* soil evaluations. This approach may have the capability to generate metabolic profiles free of inhibitory substances, such as humic acids, commonly present in *ex vivo* soil systems. In this study, we used a packed-bed microbial bioreactor to assess the role of the natural soil microbial community during biodegradation of the triazolopyrimidine fungicide, ametoctradin. Metabolite profiles produced during *in vitro* ametoctradin degradation were similar to the metabolite profiles obtained during environmental fate studies and demonstrated the degradation of 81% of the parent compound in 72 h compared to a half-life of 2 weeks when ametoctradin was left in the soil. The microbial communities of four different soil locations and the bioreactor microbiome were compared using high throughput sequencing. It was found that biodegradation of ametoctradin in both *ex vivo* soils and *in vitro* in the bioreactor correlated with an increase in the relative abundance of Burkholderiales, well characterized microbial degraders of xenobiotic compounds.

## Introduction

The human population has rapidly increased since the development of farming; however, the current growth rate is unsustainable and will eventually become limited by food demands that cannot persist using our current farming practices (Alexandratos, 1999; Tilman et al., 2002). These demands are being tackled by the scientific and agricultural communities through a collaboration set up to improve crop production, plant breeding, and disease mitigation (Tilman et al., 2002). In particular, disease management is important for maintaining food security from pests that are responsible for an annual crop loss of 5–10% (Oerke, 2006). To help combat these losses farmers have spent billions of dollars on industrialized genetically-modified (GM) plants and agrochemicals with marginal success (Klümper and Qaim, 2014; Oristep Consulting, 2015; Atwood and Paisley-Jones, 2017), as common plant pests have adapted to resist both GM plants and agrochemicals, setting in motion the need for development and testing of novel agrochemicals, which require crucial environmental fate studies to guarantee safety prior obtaining regulatory certifications.

The Federal Insecticide, Fungicide, and Rodenticide Act (FIFRA) is the most relevant regulatory statute and the compliance basis for all agrochemicals used in the United States. This regulation states that for safety before an agrochemical product can be approved for use it must be demonstrated that its residues, products of its degradation, and/or any other ingredients in the formulation do not cause adverse effects on human health and the environment (Federal Insecticide, Fungicide, and Rodenticide Act, 1910). These requirements present a unique challenge to agrochemical companies which must retrieve the agrochemical residues and products of their degradation from the soil for characterization and to show that these residues are not unreasonably harmful. This process can be costly and time-consuming, with environmental fate studies for single compounds lasting nearly a year (McDonald et al., 2006).

Currently, radiolabeling techniques coupled with advanced chemical analyses are used to determine the fate of agrochemicals in soils and plants (Soeda et al., 1972; McDonald et al., 2006). However, working with radiolabeled compounds is not always feasible, limiting residue analysis to more conventional methods such as UV detection combined with high performance liquid chromatography (HPLC-UV). An additional challenge encountered when working with soil samples arises from the presence of humic substances, which must be removed prior to the quantification of agrochemical residues, effectually raising the number of steps and the limit of detection (Masqué et al., 1998; Hogendoorn et al., 1999). Additionally, humic acids have been shown to reduce degradation efficiency *in vitro* (Zhao et al., 2018; Singh et al., 2019). Thus, *ex vivo* environmental fate and residue quantification studies could be improved by obtaining samples free of humic materials while still maintaining the sample’s biology.

While it is accepted that microorganisms provide the critical driving force behind degradation of xenobiotic compounds most studies are executed using *in vivo* (i.e., samples taken from agrochemical-treated soils at a research farm), *ex vivo* (i.e., soil cores removed from the environment and treated in the lab), and monoculture *in vitro* degradation experiments. All of these approaches present challenges such as environmental contamination, lengthy degradation times, or incomplete generation of metabolite fingerprints (Warhurst et al., 1994; van Ginkel, 1996; McDonald et al., 2006). One solution would be to use a controlled *in vitro* system to culture complex microbial consortia from soils. This strategy would facilitate the elimination of background signals coming from humic materials, allowing the rapid generation of “clean” agrochemical degradation profiles and providing detailed information regarding the specific community members responsible for actively participating in the degradation of the compound of interest. Although such a system cannot act as a replacement for field-based environmental fate studies, the metabolite profiles generated would allow for targeted downstream analyses.

In this study, we used a traditional *ex vivo* soil system as a comparison control to investigate the response of soil microbiome communities from four different locations challenged with the EPA-approved fungicide ametoctradin. Next, we validated an *in vitro* dynamic packed-bed bioreactor system (PBBR) to select for and culture complex microbial consortia capable of degrading the fungicide. This culturing technique generated a “clean” metabolite profile that was similar to the profile found in *ex vivo* soil experiments. Furthermore, we identified the microbial community members that were likely responsible for degradation of ametoctradin, subsequently making these members useful biomarkers for future field studies.

## Results

### Ametoctradin Biodegradation in an *ex vivo* Soil System

**FIGURE 1:**
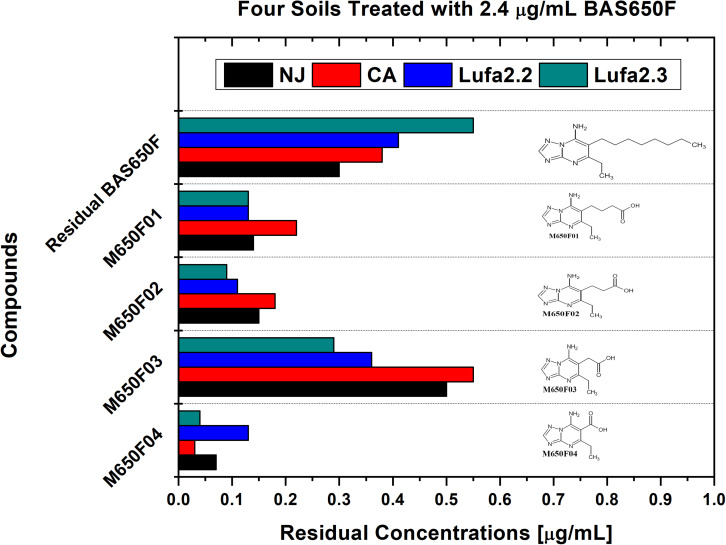
Residual concentrations of ametoctradin (BAS650F) and metabolites in soil. Residual concentrations of ametoctradin and its major metabolites in four treated soil samples. Concentrations of each compound were determined using HPLC-MS/MS. The chemical structure of each compound is shown to highlight the oxidized structure of the aliphatic side chain in the parent compound.

Soil samples were collected from four different locations previously utilized for ametoctradin environmental fate testing and regularly used for agricultural studies: California (CA), New Jersey (NJ), and Germany (LUFA 2.2 and LUFA 2.3) (Table 1). Roughly 1 kg of each soil sample was placed in two replicate containers linked to a CO_2_-scrubbed air source. Each sample was then either treated with 2.4 μg mL^–1^ of ametoctradin or remained untreated. After 14 days, the soil samples were harvested and analyzed in triplicate using HPLC-MS/MS. In each case, degradation of ametoctradin was confirmed along with the identification of four major metabolites: M650F01, M650F02, M650F03, and M650F04 (Figure 1). These compounds appeared to be the product of degradation by soil microorganisms capable of metabolizing the long aliphatic chain on the ametoctradin molecule while the amine ring structures remain largely unaltered. The NJ soil sample displayed the highest and most complete level of ametoctradin degradation, with only 13.6% of the original amount of agrochemical remaining after the 14-day period. The M650F03 metabolite appeared to be the main degradation product in every soil type.

**TABLE 1:** Soil samples used in this study.

**Soil ID**	**Location (decimal degrees)**	**Soil taxonomy**	**Shannon-Wiener index**	**Reference(s)**
CA	Tulare County, CA, United States (36.004587, −119.076090)	Nord Coarse-Loamy, Mixed, Superactive, Thermic Cumulic Haploxerolls	8.05 ± 0.0875	Khan, 2012
NJ	Hunterdon County, NJ, United States (40.533333, −74.98333)	Penn Fine-Loamy, Mixed, Superactive, Mesic Ultic Hapludalfs	7.75 ± 0.1021	Khan, 2012
LUFA 2.2	Hanhofen, Rheinland-Pfalz, Germany (49.312622, 08.327042)	Mesic Ustalfs	7.33 ± 0.1283	Khan, 2012
LUFA 2.3	Ottenbach, Rheinland-Pfalz, Germany (49.196078, 08.188425)	Mesic Udalfs	7.99 ± 0.0233	Khan, 2012

*USDA soil taxonomy is used for soils collected from the United States. The FAO soil classification is used for soils collected in Germany. Shannon-Wiener diversity index values are expressed as the average of three replicates from untreated soils.*

### Microbial Community Composition of *ex vivo* Systems

**FIGURE 2:**
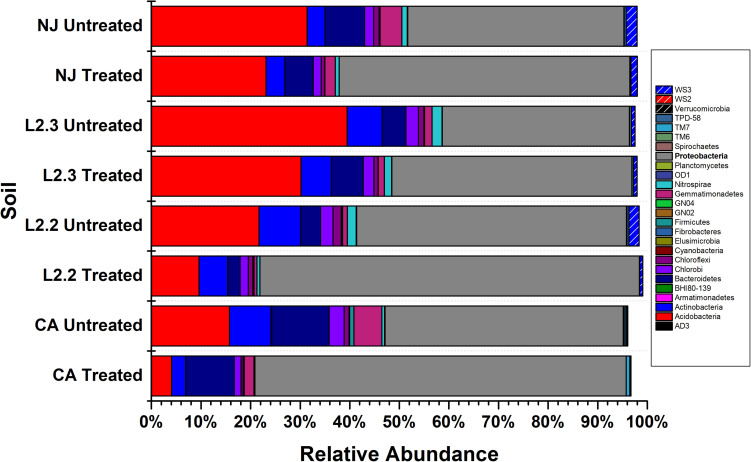
Relative abundance of bacterial phyla across soil samples. Treated soils were exposed to 2.4 μg mL^–1^ ametoctradin for 14 days. L2.2 and L2.3 represent LUFA 2.2 and LUFA 2.3 soil samples, respectively. Percent relative abundance is expressed as the mean relative abundance of three samples for each treatment.

Microbiome analysis of treated and untreated soils by 16S (Figure 2 and Supplementary Table S1) and 16S/18S (Supplementary Figure S2 and Supplementary Table S2) rRNA amplicon sequencing showed that Proteobacteria were the dominant bacterial phylum present in both the treated and untreated soils, representing up to 54% of the reads in untreated soils and 78% of the reads in treated soils. The Acidobacteria were also commonly identified, comprising up to 39% of the reads in untreated soils and 28% in treated soils. Within Eukaryotes, ametoctradin treatment only revealed significant reduction in the Oomycetes, particularly *Phytophthora infestans*, which are the target organisms for ametoctradin (Supplementary Table S2).

Species richness varied between the four soil samples, while the most diverse soils were NJ and LUFA 2.3, with Shannon-Wiener Index (SWI) values around 8. The CA and LUFA 2.2 soils had SWI values of 7.75 and 7.3, respectively (Table 1). All soils showed a significant decrease in diversity when treated with ametoctradin (CA, NJ, LUFA 2.2: *p* < 0.0001, LUFA 2.3 *p* = 0.053). Moreover, as indicated in Supplementary Table S1, several OTUs increased in relative abundance after treatment. Those OTUs that increased in relative abundance include known degraders of xenobiotic compounds such as *Mycobacterium*, *Rhodococcus*, *Comamonas*, *Burkholderia*, *Variovorax*, *Methylibium*, and *Pseudomonas*. In fact, many of these genera were below the limit of detection in untreated soils. This increase in relative abundance after soil treatment suggested the presence of resistant organisms and may be connected to microbially mediated degradation (Berg et al., 2012; Jia et al., 2015).

Soil microbial community similarities and differences have been highlighted by beta diversity analysis (Figure 6A). Although the number of samples did not allow for statistical analysis, the data showed that untreated soils tended to cluster together while treated soil sample communities tended to diverge from each other, suggesting that ametoctradin treatment on soil microbial communities varied according to the location of the soil.

### Configuration of the Packed-Bed Bioreactor System

Analysis of ex *vivo* systems showed that M650F01, M650F02, M650F03, and M650F04 were the main products of degradation of BASF650F and identified several species including *Mycobacterium*, *Rhodococcus*, *Comamonas*, *Burkholderia*, *Variovorax*, *Methylibium*, and *Pseudomonas* as relevant participants in the degradation process. We next implemented an *in vitro* continuous up-flow PBBR system to culture the complex microbial communities present in the soils in an attempt to confirm and reproduce data from *ex vivo* systems. The PBBR system consisted of a water-jacketed glass column packed with expanded glass beads. Mixing was achieved using a recirculation loop and a peristaltic pump operated at a recycling ratio of 50:1. ametoctradin was added to the medium at a maximum soluble concentration of 148 μg L^–1^. Finally, continuous addition of fresh medium containing ametoctradin was performed at five dilution rates over the course of the experiment (see section “Materials and Methods”).

### Degradation of Ametoctradin in the Packed-Bed Bioreactor

Since NJ soil samples contained the highest number of taxa and the highest diversity values, the ametoctradin-treated NJ soil was chosen to inoculate the PBBR. After inoculation, the reactor was maintained under continuous culture conditions for 1 month to permit enrichment and selection of taxa. After this maturation period, the reactor was subjected to five different dilution rates (0.03, 0.05, 0.06, 0.07, and 0.08 h^–1^) over the course of the experiment. Sequencing confirmed that no eukaryotic taxa remained in the reactor after maturation, indicating that any ametoctradin degradation observed in the reactor was entirely due to prokaryotic organisms.

**FIGURE 3:**
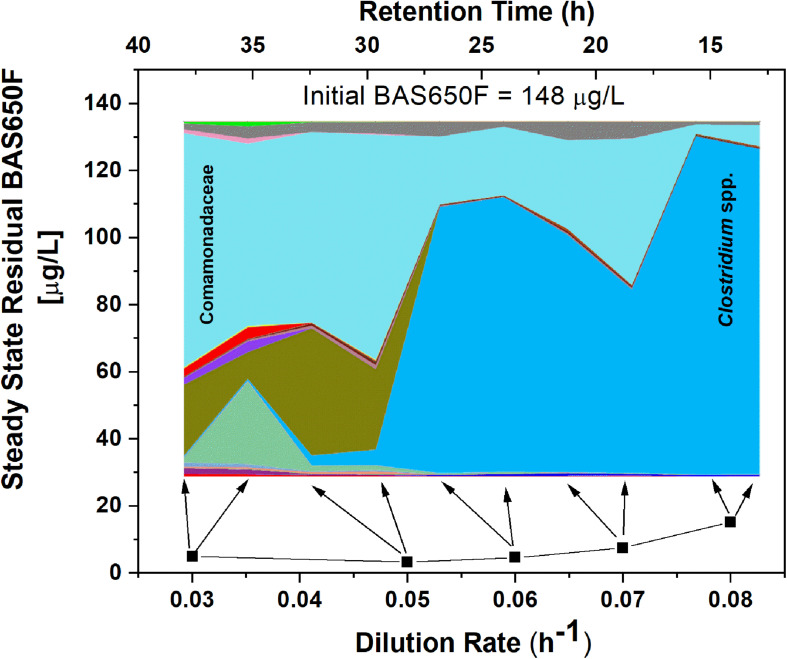
OTU relative abundance and residual ametoctradin (BAS650F) at steady-state. The area chart was generated in QIIME using OTU relative abundance data, with colors representing the relative abundance of a particular OTU. The highly abundant Comamonadaceae and *Clostridium* spp. OTUs are labeled as a reference. Residual ametoctradin levels shown are the average of two samples taken at steady-state for each respective dilution rate. Arrows indicate the two points on the area chart corresponding to these two samples at steady-state.

Once the culture reached steady-state conditions, liquid samples were taken at retention time intervals and residual levels of ametoctradin, and its degradation products, were monitored by LC-MS/MS. The community structure was also analyzed at those times by 16S rRNA amplicon sequencing. Following this period of maturation, the reactor was sequentially exposed to five sequentially increasing dilution rates (0.03, 0.05, 0.06, 0.07, and 0.08 h^–1^) of ametoctradin-containing medium over the course of the experiment. Sequencing of rRNA amplicons from samples obtained at each dilution rate confirmed the washout and absence of eukaryotic OTUs in the reactor after maturation. Nearly complete degradation of ametoctradin was observed in the reactor due to prokaryotic activity. At the lower dilution rates (0.03, 0.05, and 0.06 h^–1^), residual levels of ametoctradin were low (4.3 ± 0.89 μg L^–1^). When the dilution rate was increased to 0.07 and 0.08 h^–1^, the residual levels of ametoctradin also increased, reaching a maximum concentration of 15.2 μg L^–1^ (Figure 3). These variations in residual levels of ametoctradin matched shifts in the reactor microbial community, with the Burkholderiales, particularly *Comamonas*, dominating the community at the lower dilution rates (0.03, 0.04, and 0.05 h^–1^). A shift towards members of the Clostridiales, specifically *Clostridium*, was observed at the higher dilution rates (0.06 h^–1^ and above) (Figure 3 and Supplementary Table S3). These results indicated that Burkholderiales may be key to the ametoctradin biodegradation process.

**FIGURE 4:**
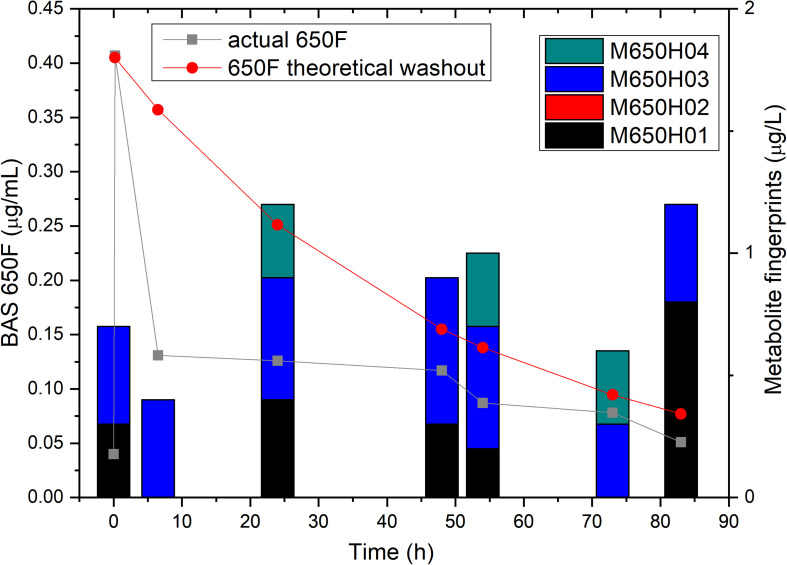
Ametoctradin (BAS650F) pulse experiment. A pulse of 0.41 μg mL^–1^ ametoctradin was performed at time 0, with samples taken immediately before and after the pulse to confirm initial levels of parent and metabolites. Samples were taken at various intervals up to 83 h post pulse. At each sampling point, levels of ametoctradin and its four major metabolites were analyzed using HPLC-MS/MS. A theoretical washout curve based on reactor parameters was calculated and plotted to confirm degradation. Residual ametoctradin levels are expressed in μg mL^–1^, while metabolite levels are expressed in μg L^–1^.

To confirm that the low residual levels of ametoctradin found in the reactor were due to microbial degradation, we performed a pulse experiment in which we quantified the levels of ametoctradin and its metabolites using LC-MS/MS at various sampling points over a period of 83 h. This data was then compared against a model for theoretical washout in which the compound would slowly be removed from the system at predictable intervals based on the dilution rate. It is important to note that the pulse of ametoctradin into the reactor was well above its limit of solubility in water, and the rapid decrease in ametoctradin levels in the first 8 h is an artifact of this limitation (Figure 4). However, increased levels of metabolites M650F01, M650F03, and M650F04 were detected in the samples, indicating active biodegradation. Additionally, the levels of ametoctradin remained relatively stable around 0.148 μg mL^–1^ (limit of solubility in water) for a period of 42 h, suggesting that the additional insoluble portion from the pulse was being solubilized as degradation of the parent compound was occurring.

### *In vitro* Composition of the New Jersey Soil-Derived Microbial Community

**FIGURE 5:**
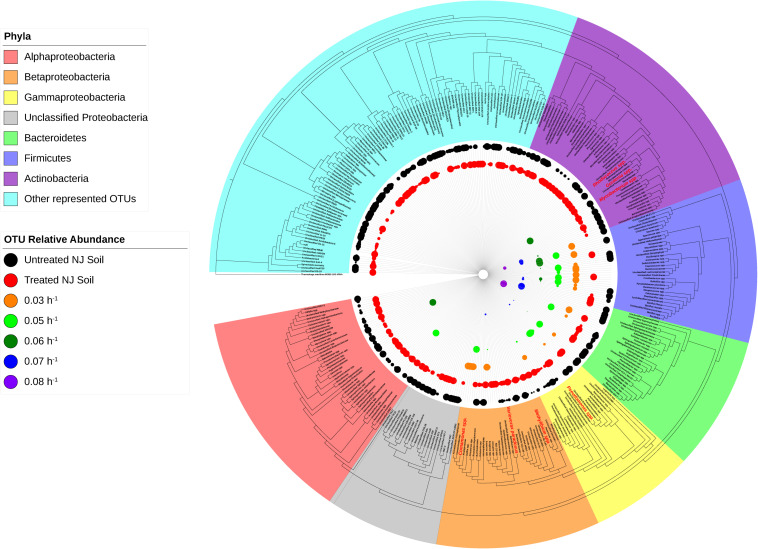
Phylogeny and relative abundance in the bioreactor and in New Jersey soil sample. Phylogenetic tree was generated in Geneious using the PHYML algorithm from the set of representative sequences generated by QIIME. The diameter of each circle is directly correlated to the percent relative abundance of the OTU, with larger circles representing higher relative abundance. OTUs with a relative abundance of <0.3% have circles of a diameter too small to be displayed. OTUs highlighted in red are well-known degraders of xenobiotic compounds.

**FIGURE 6:**
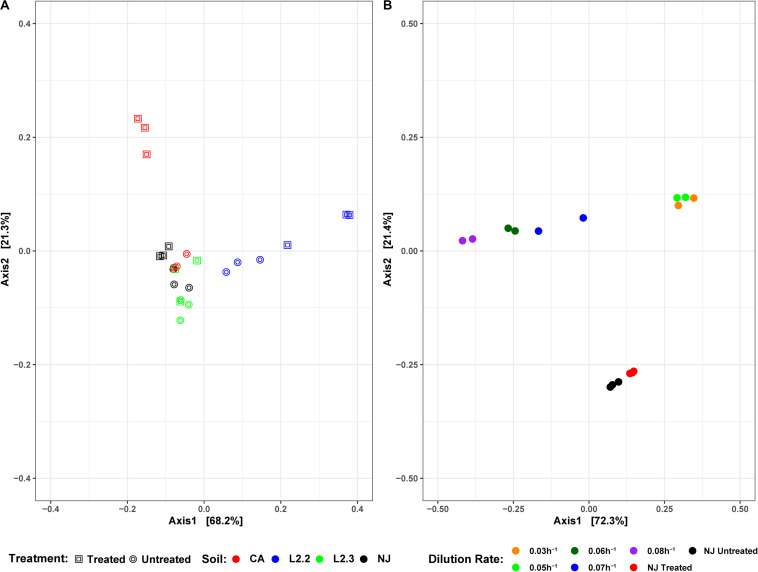
DPCoA plots for each soil sample, treatment, and reactor dilution rate. Panel **(A)** presents both the untreated and treated samples for each soil, while panel **(B)** presents the samples from each reactor dilution rate, as well as the NJ Treated and Untreated samples. Plots were generated using the vegan package in R 3.5.1. Axis 1 and Axis 2 represent principal components 1 and 2, respectively.

Microbiome analysis of the soil-derived community showed that abundance of members of the Betaproteobacteria continued to increase up to a dilution rate of 0.06 h^–1^ after which they begin to wash out of the system (Figure 5 and Supplementary Table S3). A *Comamonas* sp. was particularly abundant at these dilution rates, where it comprised nearly 60% of the reads. At higher dilution rates (0.07 and 0.08 h^–1^), it is apparent that members of the Firmicutes, particularly *Clostridium* spp., begin to dominate the microbial community in the bioreactor. Beta diversity analysis of the soil-derived communities sampled at different dilution rates showed clear differences between soil and soil-derived populations, with treated and untreated soil samples clustering separately from the PBBR samples. Likewise, samples collected from low dilution rate conditions were grouped separately from high dilution rate condition samples illustrating the dynamics of the microbial community (Figure 6). Community shifts were likely due to the changes in relative abundance in Betaproteobacteria and Clostridia detailed above. We also observed that the disappearance of Acidobacteria, a highly abundant phylum in the soil samples, in the PBBR did not hinder the ability of the consortium to degrade ametoctradin, supporting our hypothesis that Burkholderiales were the major drivers of ametoctradin degradation.

### Predicted Metabolic Pathways Impacted by Ametoctradin

**FIGURE 7:**
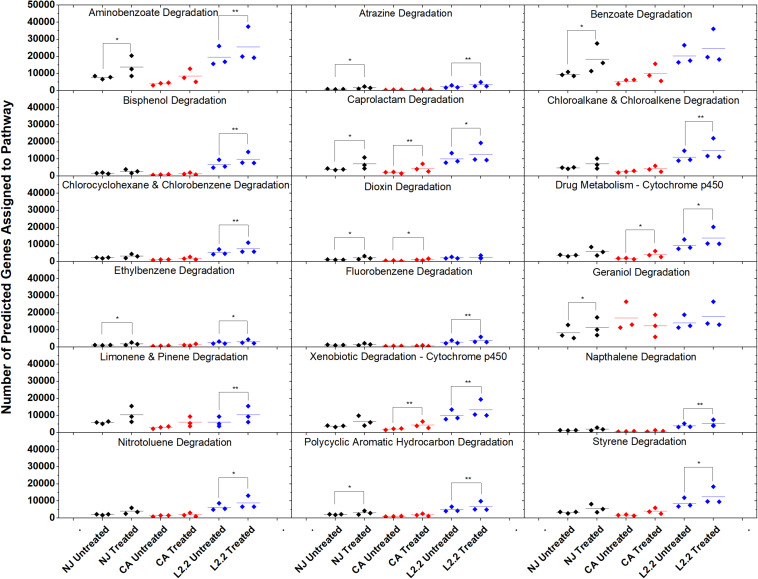
Gene counts in KEGG pathways associated with xenobiotic degradation in soils. Gene counts are determined in PICRUSt by first finding sequenced representatives for each OTU, summing the number of genes involved in a pathway for that OTU, multiplying this number by the relative abundance of the OTU, and finally by summing across OTUs. Boxplots were created in Origin 9.1 by pooling three replicates for each soil and treatment. Statistics on metagenome profiles were performed in STAMP. Each sample is represented with a diamond. Colored lines represent the mean of the three samples. Brackets with a single asterisk correspond to a *p*-value of <0.05, while two asterisks correspond to a *p*-value of <0.01.

Using the predictive software PICRUSt, which permits the determination of metabolic potential from 16S rRNA amplicon sequencing data, we analyzed the KEGG gene pathways associated with degradation of xenobiotic compounds in *ex vivo* and *in vitro* systems (Figure 7). The only pathway that showed a significant difference in relative abundance between treatments in all three soils was caprolactam degradation. This pathway contains several enzymes that may participate in the oxidation of the aliphatic side chain present on ametoctradin, including alkane 1-monoxygenase (EC 1.14.15.3), NADP-dependent aldehyde dehydrogenase (EC 1.2.1.4), and NADP-alcohol dehydrogenase (EC 1.1.1.2). The LUFA 2.2 soil showed the largest number of changes in metabolic potential between non-treated and treated samples, with 15 out of 18 pathways examined showing significantly higher gene counts in the treated soil. Specifically, gene predictions related to bisphenol, chloroalkane, fluorobenzene, naphthalene, and styrene degradation only showed significant increases in the LUFA 2.2 soil. Several of these pathways include decyclizing enzymes such as catechol dioxygenase (ECs 1.13.11.1 and 1.13.11.2) which might participate in the degradation of the ring structure in ametoctradin.

**FIGURE 8:**
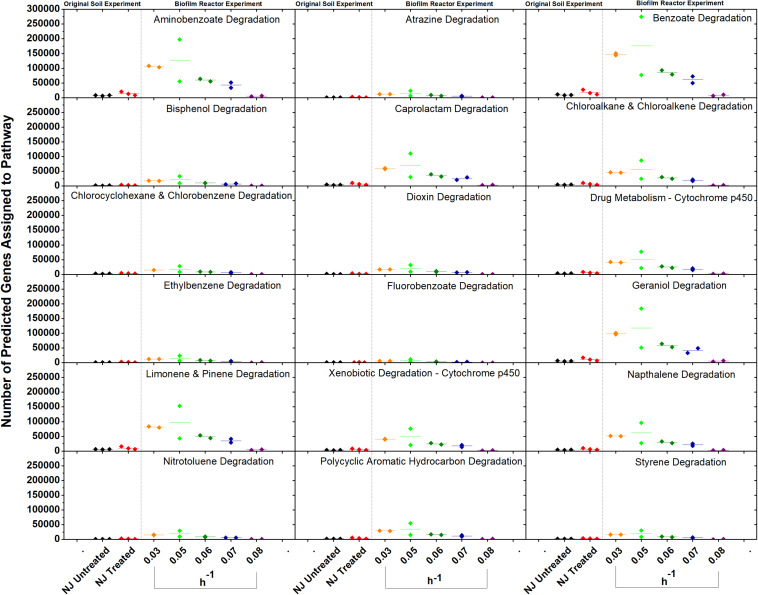
Gene counts in KEGG pathways associated with xenobiotic degradation in the bioreactor. Gene counts are determined in PICRUSt by first finding sequenced representatives for each OTU, summing the number of genes involved in a pathway for that OTU, multiplying this number by the relative abundance of the OTU, and finally by summing across OTUs. Boxplots were created in Origin 9.1 by pooling three replicates for each soil and two replicates for each dilution rate. A dashed line separates the soil and bioreactor experiments in each panel to stress the fact that each graph contains data from two separate experiments. Dilution rates are expressed in h^–1^.

*In vitro* analysis of changes in metabolic potential showed that the dilution rates impacted a number of genes for each category that were directly correlated with changes in the relative abundance of Betaproteobacteria, specifically a *Comamonas* species (Figure 8). Upon examination of the *Comamonas* genomes in the KEGG database, we found that all five annotated genomes contained decyclizing dioxygenases such as catechol dioxygenase (ECs 1.13.11.1 and 1.13.11.2) and protocatechuate dioxygenase (EC 1.13.11.8) (Kanehisa and Goto, 2000). This abundance of ring-opening enzymes may help to explain the low amounts of ametoctradin and its metabolites in the reactor effluent.

## Discussion

This study examined the ability of an *in vitro* dynamic PBBR to simulate agrochemical metabolite degradation profiles found in *ex vivo* soil experiments. Additionally, we analyzed the microbiome of both soil and bioreactor samples to identify the members of the microbial community who responded to treatment with an EPA-approved agrochemical, ametoctradin, and potentially participated in its degradation.

Notably, the packed-bed reactor system was able to accelerate the degradation of ametoctradin. Packed-bed bioreactors have been used for decades for microbial biodegradation of environmental contaminants and compounds of interest, as well as for the production of value-added products such as ethanol and lactic acid (Shiotani and Yamané, 1981; Strandberg et al., 1989; Bruno-Bárcena et al., 1999; Alfonso-Gordillo et al., 2016; Geed et al., 2017; Mavriou et al., 2020). It has been shown previously that sessile microbial cells are more resistant to toxic substances than planktonic cells, making packed-bed bioreactors attractive for studying the degradation of xenobiotic compounds (Parkin and Speece, 1983). Additionally, packed-bed bioreactors have several advantages over stirred-tank bioreactors including compactness, resistance to temperature fluctuations, and limited biomass washout (Sagberg et al., 1992). In the soil, this compound exhibits a half-life of approximately 14 days, whereas 81% of the compound had degraded in 72 h in the bioreactor. Furthermore, 3 of the 4 major metabolites were detected in small amounts in each of the samples, indicating rapid assimilation or mineralization to CO_2_ (Figure 4). Additionally, the culturing medium was free of compounds that may interfere with the analysis of ametoctradin and its metabolites, allowing for a simple and rapid downstream analysis.

The degradative ability of the members of the Burkholderiales has been well documented in the literature (Pérez-Pantoja et al., 2012). Our data demonstrated that members of the Burkholderiaceae and Comamonadaceae, including *Comamonas*, *Variovorax*, and *Methylibium*, are highly abundant in samples that showed evidence of ametoctradin degradation. These species have been well studied and are commonly associated with xenobiotic degradation (Vallaeys et al., 1998; Nakatsu et al., 2006; Satola et al., 2012; Chen et al., 2016a, b; Öztürk et al., 2020; Zhang et al., 2020). In the packed-bed reactor, *Comamonas* spp. account for nearly 60% of sequence reads at the dilution rates 0.03 and 0.05 h^–1^ (Supplementary Table S3). The high levels of *Comamonas* spp. at these dilution rates also coincides with lower residual levels of ametoctradin in the reactor effluent, likely indicating that *Comamonas* is largely responsible for ametoctradin degradation in the reactor (Figure 3). Additionally, several Actinomycetes, including *Gordonia*, *Mycobacterium*, and *Rhodococcus* were identified in soil and bioreactor samples via their 16S rRNA gene sequences (Figure 5 and Supplementary Tables S1, S3). These genera have been extensively studied and have been shown to be excellent degraders of various xenobiotic compounds (Burback and Perry, 1993; Arenskötter et al., 2004; Chen et al., 2016b). Interestingly, an unclassified OTU from the family Lachnospiraceae, a part of the Clostridia, was highly abundant in the 0.03 and 0.05 h^–1^ conditions, comprising 25 and 30% of reads, respectively (Supplementary Table S3). There are reports of Lachnospiraceae degrading hydrocarbons (Schouw et al., 2016), however, this family are mainly known for their cellulolytic activity in ruminants (Zhang et al., 2017). Considering that cellulosic compounds are present in the culture medium and that ruminant manure is a common fertilizer in agriculture, this Lachnospiraceae group is likely not contributing to ametoctradin degradation.

Furthermore, we examined the impact of ametoctradin treatment on archaea and eukaryotes, since it is well understood that fungi play a noteworthy role in the biodegradation of xenobiotic compounds (Bending et al., 2002; Gupta et al., 2017; Manasfi et al., 2020). Overall, these populations were quite stable, with significant changes only occurring in the oomycetes, which are the target organisms of ametoctradin (Supplementary Figure S2 and Supplementary Table S2). No archaeal or eukaryotic sequences were detected in the bioreactor, indicating that bacteria mediated all degradation occurring therein.

Our PICRUSt-generated metagenomes also indicated the possible presence of genes associated with xenobiotic degradation, including monooxygenases and dioxygenases, hydrolases, cytochromes p450 and more, many of which are produced by microorganisms found in our packed-bed reactor, such as *Comamonas* (Rhee et al., 2004). Due to the predicted presence of many of these genes, this system would likely be useful in examining the microbial degradation of many different classes of xenobiotic compounds. A packed-bed system would also be ideal for examining the ability of a defined microbial consortium to degrade various other xenobiotic compounds in a controlled environment while also allowing for complete compound accountability.

Here, we demonstrated the ability of a naturally sourced microbial consortium to degrade the triazolopyrimidine fungicide ametoctradin and generate metabolite profiles *in vitro*. Additionally, we presented a novel usage of a PBBR that facilitated cleaner downstream analysis of agrochemical degradation. This system may also provide researchers with future opportunities for a more intimate analysis of microbial community dynamics in the degradation of xenobiotic compounds.

## Materials and Methods

### Collection and Treatment of Soil Samples

Two soil samples from the United States and two samples from Germany were collected for *ex vivo* degradation analysis (Table 1). Each of the four soil samples were treated with 2.4 mg kg^–1^ soil of ametoctradin and allowed to react for a total of 14 days. After this incubation period, the soils were analyzed following established methods for residual ametoctradin and its associated metabolites (M650F01, M650F02, M650F03, M650F04) using HPLC-UV and HPLC-MS/MS, respectively (European Food Safety Authority, 2012).

### Sequencing of Soil and Reactor Samples

Amplification of the V1–V2 and V3–V4 regions of the bacterial 16S rRNA gene were performed on total DNA from soil samples. Master mixes for these reactions used the Qiagen HotStar Hi-Fidelity Polymerase Kit (Qiagen, Valencia, CA, United States) with a forward primer composed of the Roche Titanium Fusion Primer A (5′-CCATCTCATCCCTGCG TGTCTCCGACTCAG-3′), a 10 bp Multiplex Identifier (MID) sequence (Roche, Indianapolis, IN, United States) unique to each of the samples. The gene-specific part of the primer used the universal bacterial primer 8F (5′-AGAGTTTGATCCTGGC TCAG-3′) to amplify V1–V2 region or 515 F bacterial primer (5′-GTGCCAGCMGCCGCGGTAA-3′) to amplify V3–V4 region (Edwards et al., 1989; Fierer et al., 2008; Caporaso et al., 2011; Walters et al., 2016). The reverse primer was composed of the Roche Titanium Primer B (5′-CCTATCCCCTGT GTGCCTTGGCAGTCTCAG-3′), the identical 10 bp MID sequence as the forward primer and the reverse bacterial primer 338R (5′-GCTGCCTCCCGTAGGAGT-3′), which span the V1–V2 hyper variable region of the bacterial 16S or 806R bacterial primer (5′-GGACTACHVGGGTWTCTAAT-3′) for the amplification of V3–V4 region. The thermal profile for the amplification of both V1–V2 and V3–V4 regions had an initial denaturing step at 94°C for 5 min, followed by a cycling of denaturing of 94°C for 45 s, annealing at 50°C for 30 s and a 1 min 30 s extension at 72°C (35 cycles), a 10 min extension at 72°C and a final hold at 4°C. Each sample was individually gel purified using the Qiaquick Gel Extraction Kit (Qiagen, Valencia, CA, United States) and E-Gel Electrophoresis System (Life Technologies, Invitrogen division), and standardized prior to pooling. Pooled barcoded libraries were sequenced on a Roche 454 Genome Sequencer FLX Titanium instrument (Microbiome Core Facility, Chapel Hill, NC, United States) using the GS FLX Titanium XLR70 sequencing reagents and protocols. Initial data analysis, base pair calling and trimming of each sequence to yield high quality reads were performed by Research Computing at the University of North Carolina at Chapel Hill (Chapel Hill, NC, United States). Sequence data were cleaned and analyzed using Version 1.9 of QIIME software (Caporaso et al., 2010). Sequences are available on the NCBI GenBank under SRA accession number PRJNA625701.

### Building and Annotating Phylogenetic Trees

Redundant sequences were removed from the set of representative sequences using the PhyloToAST (Dabdoub et al., 2016) plugin for QIIME. The trimmed set of representative sequences was aligned using the MUSCLE algorithm in Geneious 5.5.9 (Biomatters Inc., Newark, NJ, United States). Maximum likelihood phylogenetic trees were generated using the PHYML plugin for Geneious. Sequence ID numbers on leaves were replaced with taxon information using PhyloToAST (Dabdoub et al., 2016). Trees were annotated with relative abundance information using the Interactive Tree of Life (iToL) software (Letunic and Bork, 2011). Relative abundance data were generated by QIIME and normalized using the cumulative sum scaling (CSS) method and the normalize_table.py command in QIIME for each project (Paulson et al., 2013). Mean relative abundance was calculated from the mean of the triplicate data points for each sample and repeated for each of the different treatments using PhyloToAST.

### Construction and Analysis of Metagenomic Profiles

In order to obtain a rough metagenome for each soil treatment and bioreactor condition, we ran PICRUSt analysis (Langille et al., 2013). The input file used in PICRUSt was a closed-reference OTU table generated for both the soil and bioreactor experiments in QIIME. Predicted metagenomes were statistically analyzed in STAMP (Parks et al., 2014).

### Bioreactor Enrichment Experiment

The bioreactor enrichment experiment was performed in a packed-bed reactor consisting of a single water-jacketed glass column (6 cm diameter by 13 cm height) filled with porous foam glass particles having a mean diameter of 4–8 mm with a porosity of 55%, and a density of ∼0.2 g cm^–3^ (A generous gift from Poraver GmbH). The column had an external loop that consisted of an input for the growth medium, a peristaltic pump controlling the dilution rate, a port from which to take samples, and a harvest tank and associated peristaltic pump. A schematic of the PBBR configuration can be found in the Supplementary Figure S3. The reactor was fed with medium containing 148 μg L^–1^ ametoctradin (maximum solubility in water); a full description of medium composition can be found in the Cultivation Medium section “Materials and Methods” below. This medium was recycled within the reactor at ratio of 50:1. The final active volume for the duration of the experiment was 220 mL. The temperature during the experiment was controlled via water jacket at 24°C. The pH was monitored but not controlled and varied between 4.8 and 6.7. Inoculum for the experiment consisted of 1 g of ametoctradin treated NJ soil. After inoculation, the *in vitro* microbial community was allowed to mature and stabilize for 1 month at a dilution rate of 0.03 h^–1^ before adjustments were made to the dilution rate. Over the next 3 weeks the reactor’s microbial community was subjected to five different sequential dilution rates (0.03, 0.05, 0.06, 0.07, and 0.08 h^–1^). For each dilution rate, performed for at least a three retention times (RT) (period of adaptation), three samples were taken and stored for sequencing and HPLC-UV or HPLC-MS/MS analysis. The samples represent the mature microbial community that has been influenced by the presence of ametoctradin.

### Ametoctradin Pulse Experiment

To determine if residual levels of ametoctradin and products of its degradation in the bioreactor were microbially mediated degradation and not washout, an experiment injecting 0.41 μg mL^–1^ ametoctradin was performed while maintaining the reactor at a dilution rate of 0.03 h^–1^. Samples were taken at 0.2, 6.5, 24, 48, 54, 73, and 83 h to monitor levels of ametoctradin and its metabolites. Actual data analyzed by HPLC-UV or HPLC-MS/MS were compared to theoretical values obtained from a mathematical model for washout at a given dilution rate. The formula for theoretical substrate concentration at time *t* [(*Y*)*_*t*_*] is as follows:

$${\left(Y\right)}_{t}=\left(\frac{C_{0}\times V_{0}}{V_{R}}\right)\times e^{\left(-t\times D\right)}$$

where *C*_0_ and *V*_0_ represent the initial pulse concentration and volume, *V*_*R*_ is the total reactor volume, and *D* is the reactor dilution rate.

### Culture Media

The medium used during these experiments contained (g L^–1^) yeast extract 1, ammonium acetate 2, carboxymethyl cellulose 2, dextrose 1, cellobiose 0.1, xylose 0.771, K_2_HPO_4_ 0.8, KH_2_PO_4_ 0.2, CaCl_2_⋅H_2_O 0.09, FeSO_4_⋅7H_2_O 0.0028, MgSO_4_⋅7H_2_O 0.2, MnSO_4_⋅H_2_O 0.003, NaCl 0.1, NaMoO_4_⋅2H_2_O 0.00025, ZnSO_4_⋅7H_2_O 0.00025, Biotin 0.0001, Pyridoxol⋅HCl 0.0002, and 0.01 mM EDTA.

## Data Availability Statement

The datasets presented in this study can be found in online repositories. The names of the repository/repositories and accession number(s) can be found below: https://www.ncbi.nlm.nih.gov/bioproject/PRJNA625701.

## Author Contributions

HW, MA-P, and JB-B wrote and interpreted the data in the manuscript. JB-B and MA-P designed and executed the experiments. CT and MS analyzed using HPLC-UV or HPLC-MS/MS the samples presented in the manuscript. All authors contributed to the article and approved the submitted version.

## Conflict of Interest

CT and MS were employed by the BASF Corporation. The remaining authors declare that the research was conducted in the absence of any commercial or financial relationships that could be construed as a potential conflict of interest.

## Supplementary Material

FIGURE S1 Phylogeny and relative abundance of bacterial sequences in each of the four soils examined. Phylogenetic tree was generated in Geneious using the PHYML algorithm from the set of representative sequences generated by QIIME. The diameter of each circle is directly correlated to the percent relative abundance of the OTU, with larger circles representing higher relative abundance. OTUs with a relative abundance of <0.3% have circles of a diameter too small to be displayed. OTUs highlighted in red are well-known degraders of xenobiotic compounds.

FIGURE S2 Phylogeny and relative abundance of archaeal and fungal sequences in each of the four soil samples examined. Phylogenetic tree was generated in Geneious using the PHYML algorithm from the set of representative sequences generated by QIIME. The diameter of each circle is directly correlated to the percent relative abundance of the OTU, with larger circles representing higher relative abundance. OTUs with a relative abundance of <0.3% have circles of a diameter too small to be displayed. The Oomycetes, highlighted by the black box, are the target organisms of ametoctradin.

FIGURE S3 A schematic of the packed-bed bioreactor. Arrows indicate the direction of flow through the system. The speed of the harvest pump and the recirculation pump were constant, while the speed of the feed pump was varied according to the dilution rate examined.
