# Supplementary figures and images for: Accelerated Biodegradation of the Agrochemical Ametoctradin by Soil-Derived Microbial Consortia
Source: Front Microbiol. 2020 Aug 25;11:1898. doi: 10.3389/fmicb.2020.01898 (PMC7477900; doi:10.3389/fmicb.2020.01898)

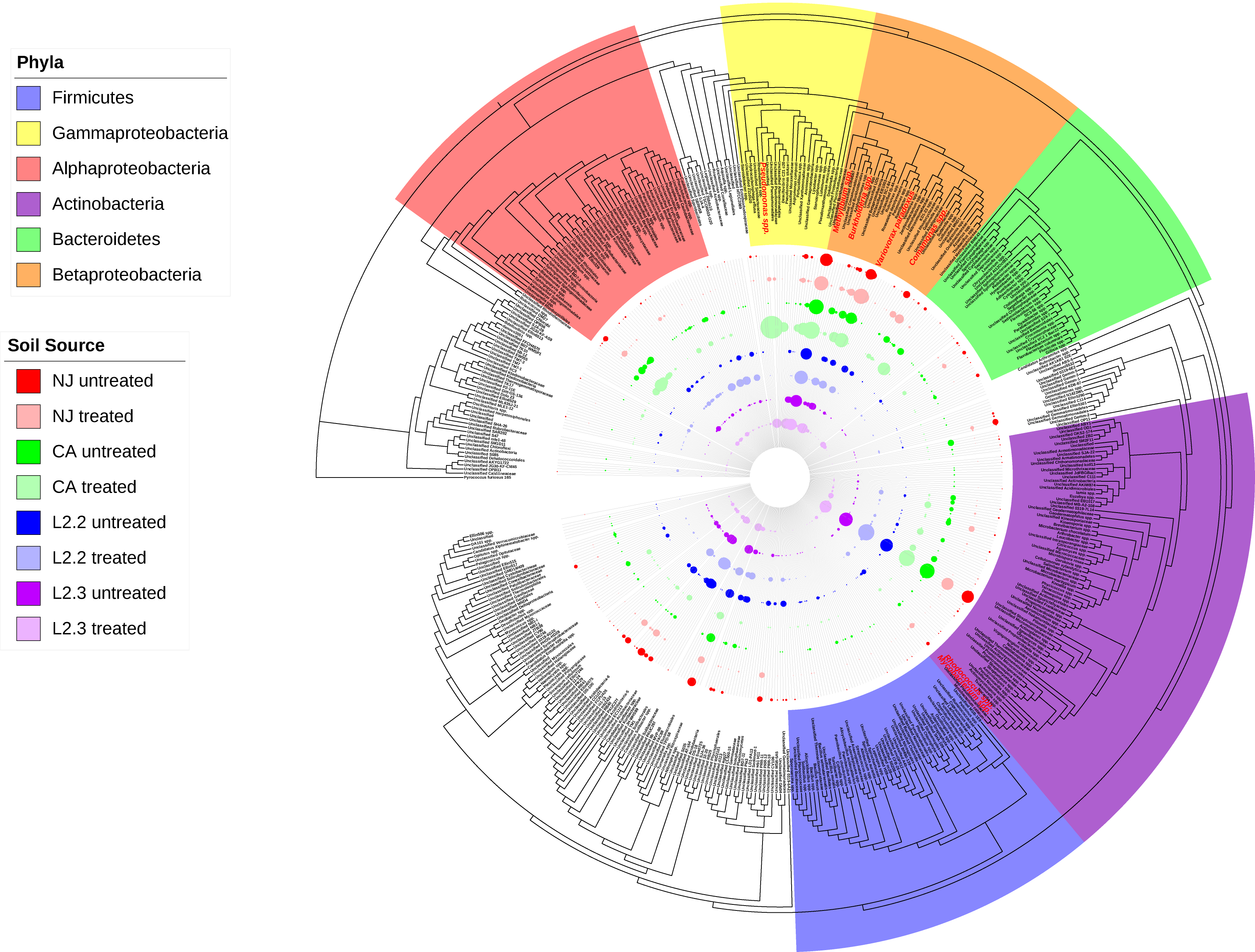

Supplement: FIGURE S1 — Phylogeny and relative abundance of bacterial sequences in each of the four soils examined. Phylogenetic tree was generated in Geneious using the PHYML algorithm from the set of representative sequences generated by QIIME. The diameter of each circle is directly correlated to the percent relative abundance of the OTU, with larger circles representing higher relative abundance. OTUs with a relative abundance of <0.3% have circles of a diameter too small to be displayed. OTUs highlighted in red are well-known degraders of xenobiotic compounds. [file Image_1.TIF]

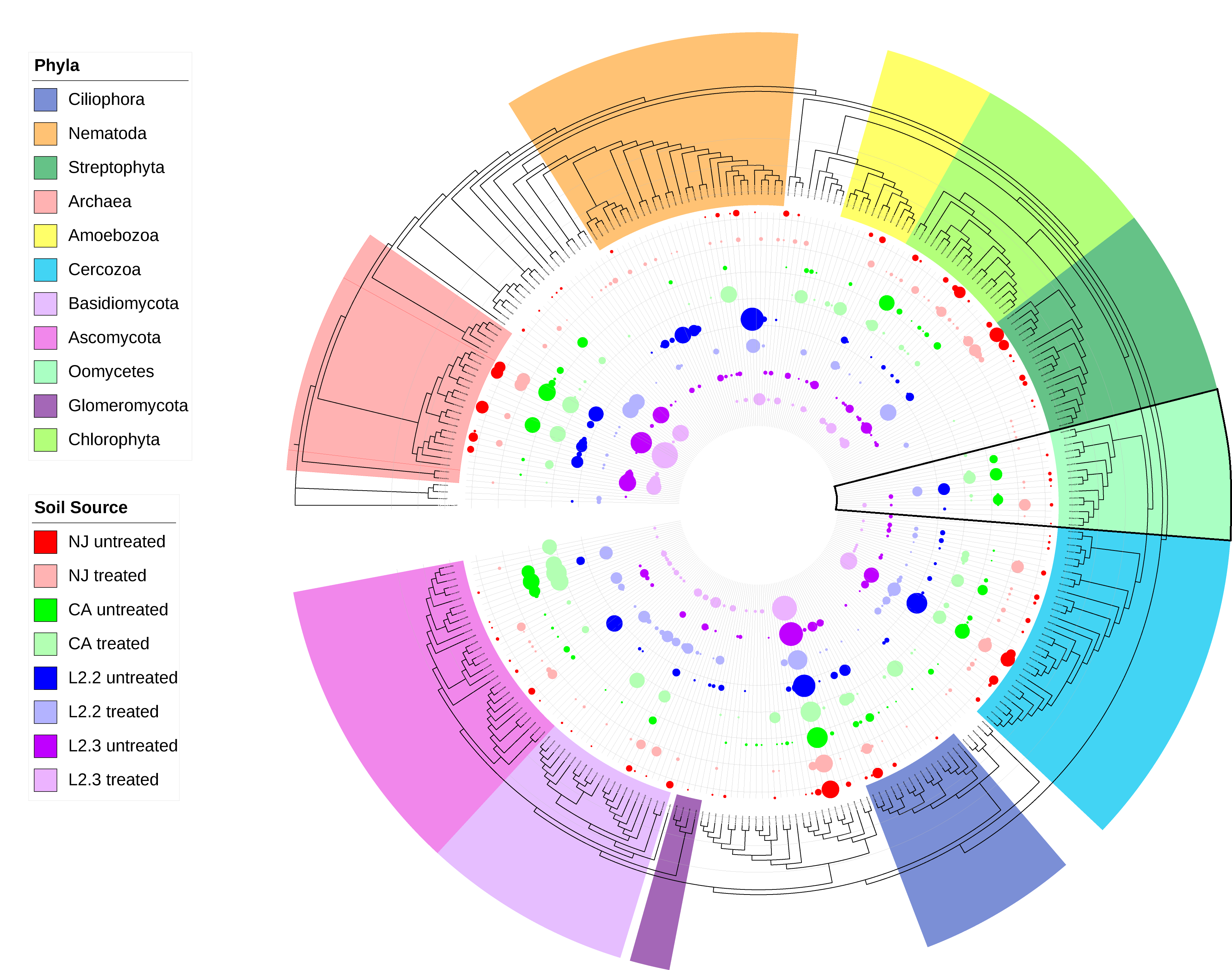

Supplement: FIGURE S2 — Phylogeny and relative abundance of archaeal and fungal sequences in each of the four soil samples examined. Phylogenetic tree was generated in Geneious using the PHYML algorithm from the set of representative sequences generated by QIIME. The diameter of each circle is directly correlated to the percent relative abundance of the OTU, with larger circles representing higher relative abundance. OTUs with a relative abundance of <0.3% have circles of a diameter too small to be displayed. The Oomycetes, highlighted by the black box, are the target organisms of ametoctradin. [file Image_2.TIF]

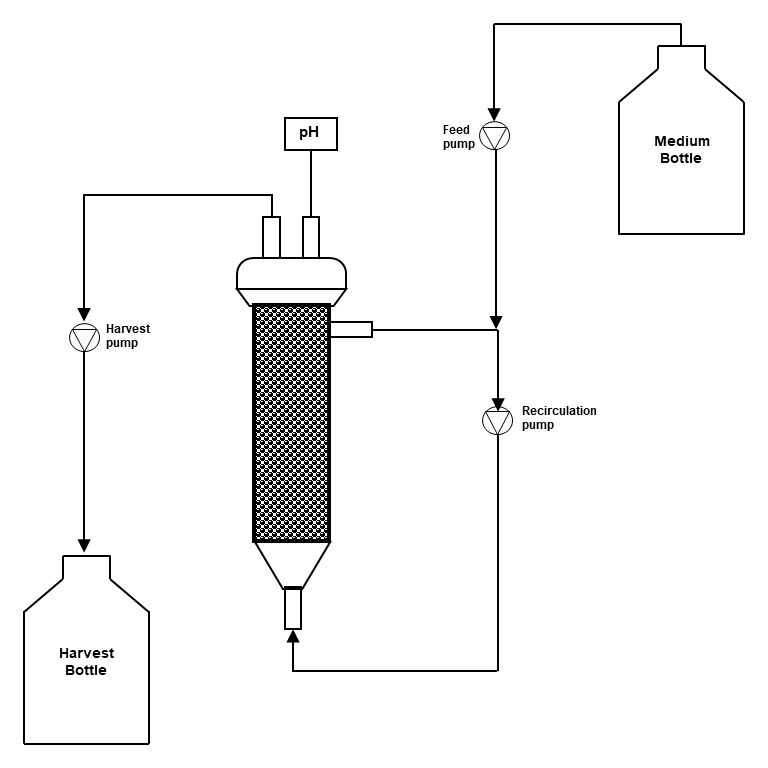

Supplement: FIGURE S3 — A schematic of the packed-bed bioreactor. Arrows indicate the direction of flow through the system. The speed of the harvest pump and the recirculation pump were constant, while the speed of the feed pump was varied according to the dilution rate examined. [file Image_3.TIF]
